# Computer Simulation of Hydrodynamic and Thermal Processes in DLD Technology

**DOI:** 10.3390/ma14154141

**Published:** 2021-07-25

**Authors:** Gleb A. Turichin, Ekaterina A. Valdaytseva, Stanislav L. Stankevich, Ilya N. Udin

**Affiliations:** World-Class Research Center, “Advanced Digital Technologies”, State Marine Technical University, 190121 Saint Petersburg, Russia; s.stankevich@ilwt.smtu.ru (S.L.S.); youdin@ilwt.smtu.ru (I.N.U.)

**Keywords:** direct laser deposition, heat transfer, mass transfer, hydrodynamics, simulation of the melt pool, alloys

## Abstract

This article deals with the theoretical issues of the formation of a melt pool during the process of direct laser deposition. The shape and size of the pool depends on many parameters, such as the speed and power of the process, the optical and physical properties of the material, and the powder consumption. On the other hand, the influence of the physical processes occurring in the material on one another is significant: for instance, the heating of the powder and the substrate by laser radiation, or the formation of the free surface of the melt, taking into account the Marangoni effect. This paper proposes a model for determining the size of the melt bath, developed in a one-dimensional approximation of the boundary layer flow. The dimensions and profile of the surface and bottom of the melt pool are obtained by solving the problem of convective heat transfer. The influence of the residual temperature from the previous track, as well as the heat from the heated powder of the gas–powder jet, taking into account its spatial distribution, is considered. The simulation of the size and shape of the melt pool, as well as its free surface profile for different alloys, is performed with 316 L steel, Inconel 718 nickel alloy, and VT6 titanium alloy

## 1. Introduction

The direct laser deposition (DLD) process, according to the classification considered in [1], is currently becoming a more and more promising technology for the additive production of parts for various purposes in shipbuilding, aircraft construction, mechanical engineering, and other industries. There is already a positive experience in the manufacture of ship fittings, propellers [2,3], water jet propellers [4,5], large-sized products and machine parts [6], high-pressure vessels, and others. This technology belongs to the direct metal deposition (DMD) technologies, in which the product is formed from a metal powder supplied by a gas jet directly into the area of action of focused laser radiation. In this case, the heating and melting of the powder and the substrate is controlled by equipment in order to maintain the process in the stability zone. The high power of the laser source should ensure and maintain a high melting rate of the powder in order to ensure high process productivity up to 1.5–2.5 kg/h [4]. However, this can lead both to the appearance of instability of the wall formation described in [7], and to a deviation from the specified dimensions [8]. A large number of important parameters of the processing mode, and the danger of leaving the process in the zone of unstable formation, greatly complicate the selection of technological parameters by the experimental method. Therefore, the availability of a physically adequate and fast mathematical model that allows the performance of numerical modeling will facilitate the development and optimization of technological parameters for the direct laser deposition process. A large number of papers describe the use of various numerical schemes of finite element analysis [9,10,11], analytical models [12], and even statistical models [13] for modeling thermal and hydrodynamic processes. However, in most of them, the case of cladding the bead on a thick and wide substrate is considered. This is not suitable for thin walls, due to differences in boundary conditions. The influence of the Marangoni effect, capillary forces, and the mutual influence of hydrodynamics and heat transfer in the melt pool are also rarely taken into account. Finite element analysis allows scientists to take these factors into account, but the calculation time can be hours, or even days. The presented work is a development of the model developed earlier in [6,7,14,15]; it was developed specifically for modeling the process of forming thin-walled structures. This model takes into account the Marangoni effect, the transfer of the powder by a gas jet, the heating of the powder by laser radiation, the interaction of the jet with the substrate, and heat transfer in the solid and liquid phases, as well as the hydrodynamics of the melt pool. This work presents the results of theoretical studies and modeling of joint thermal and hydrodynamic processes in the stationary case for the DLD process, taking into account the influence of the heated powder on the melt pool.

## 2. Materials and Methods

### 2.1. Melt Flow Description

**Figure 1:**
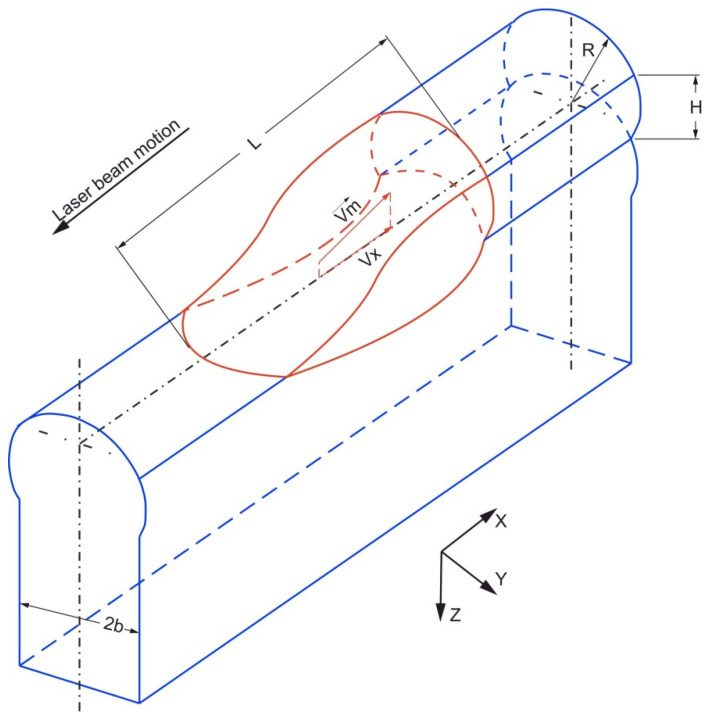
Design diagram of the deposited wall and the melt pool.

The high-speed DLD process is characterized by the formation of a melt pool with a length “L” much larger than the width “b” and depth “H” (Figure 1).

In this case, to describe the velocity field in the melt, we can limit ourselves to a one-dimensional formulation, and use the approximation of a one-dimensional boundary layer. In this case, the longitudinal velocity *V_x_*, directed along the direction of movement of the laser, is much greater than the transverse velocities *V_y_* and *V_z_*. In the case of a steady-state process, the Navier–Stokes fluid motion equation can be written as:

(1) $$V_{x}\frac{\partial V_{x}}{\partial x}=-\frac{1}{\rho }\frac{\partial p}{\partial x}+\nu \frac{\partial^{2}V_{x}}{\partial z^{2}}$$

The boundary condition at the “bottom” of the melt pool is given as:

$${V_{x}|}_{z=0}=0,$$

The boundary condition on the “top” surface is obtained from the requirement of the stress tensor continuity:

$${-\eta \frac{\partial V_{x}}{\partial z}|}_{z=H}=\frac{\partial \sigma }{\partial x}$$

We assume that the temperature change along the melt pool surface is much less than the average temperature of its surface. We assume that *T_s_* is the maximum surface temperature, while *T_m_* and *T_b_* are the melting and evaporation temperatures, respectively. Assuming that the law oftemperature drop to the tail of the melt pool is linear , then we can write:

(2) $${\eta \frac{\partial V_{x}}{\partial z}|}_{z=H}=\frac{\sigma }{L}\frac{T_{s}-T_{m}}{T_{b}-T_{m}}=\frac{\sigma^{*}}{L}$$

Let the velocity distribution of the liquid in the melt have a parabolic shape. This approach will ensure that the boundary conditions and the conditions of mass flow conservation along the “x” axis are met.

(3) $$V_{x}\left(z\right)=V_{x}\left(\alpha +\beta z+\gamma z^{2}\right)$$

Substituting Equation (3) into the boundary conditions, we obtain expressions for the coefficients of the equation:

(4) $$\begin{array}{l} \alpha =0\\ \gamma =-\frac{3}{4H}\left(\frac{2}{H}-\frac{\sigma^{*}}{L\eta V_{x}}\right)\\ \beta =\frac{2}{H}\left(1+\frac{H}{4}\left(\frac{2}{H}-\frac{\sigma^{*}}{L\eta V_{x}}\right)\right)=\frac{2}{H}\left(\frac{3}{2}-\frac{\sigma^{*}}{4\eta V_{x}}\frac{H}{L}\right)=\frac{3}{H}-\frac{\sigma^{*}}{2\eta V_{x}L} \end{array}$$

Then Equation (1) will look like this:

(5) $$V_{x}\frac{\partial V_{x}}{\partial x}=-\frac{1}{\rho }\frac{\partial p}{\partial x}-3\nu \frac{V_{x}}{H^{2}}+\frac{3\sigma^{*}}{2\rho LH}$$

We will use the continuity equation to relate the position of the weld pool surface to the melt velocity *V_x_*. Here, it is necessary to take into account that the density of the mass flow *j*(*x*) incident on the melt surface is determined by a gas–powder jet. Then the continuity equation for the flow will be written as follows:

(6) $$\frac{\partial }{\partial x}\left(V_{x}H\right)=\frac{j\left(x\right)}{\rho }$$

Considering that the pressure in the melt:

$$p=\frac{\sigma }{R}$$

and when *H < b* we get:

$$R\approx b+\frac{H^{2}}{2b},\;p\approx \frac{\sigma }{b}-\frac{\sigma H^{2}}{2b^{3}}$$

In this case, the terms of the Navier–Stokes equation associated with the change in the transverse radius of curvature of the surface will look like:

$$\frac{\partial p}{\partial x}=\frac{\sigma }{2b^{3}}\frac{H\partial H}{\partial x}$$

A change in the longitudinal radius of the curvature of the melt surface will give an additional pressure, which can be expressed as:

$$p_{add}=\sigma \frac{\partial^{2}H}{\partial x^{2}}.$$

Then, one can write:

(7) $$V_{x}\frac{\partial V_{x}}{\partial x}=\frac{\sigma }{\rho b^{3}}\frac{H\partial H}{\partial x}-3\nu \frac{V_{x}}{H^{2}}+\frac{3\sigma^{*}}{2\rho LH}$$

After integration, we will get:

(8) $$H\left(x\right)=\frac{1}{\rho v_{x}}\int_{0}^{x}j\left(x\right)dx$$

The boundary conditions for the last equation can be written as:

$${H|}_{x=0}=0,\;{\frac{\partial H}{\partial x}|}_{x=0}=0,\;{\frac{\partial H}{\partial x}|}_{x=L}=0.$$

Using Equation (8) in Equation (7), and solving it numerically, we obtain the profile of the upper surface of the melt pool, taking into account the Marangoni effect. The parameters of the length “L” and the depth “H” of the melt pool are determined by the solution to the heat transfer problem.

### 2.2. Influence of the Powder Jet on the Heat Transfer in the Deposited Wall

As was shown in [6], the thermal field when applying a single deposited bead to a thin wall in a steady state can be described by the equation of convective heat transfer. At the same time, since the wall width is comparable to the diameter of the laser beam spot, the temperature gradient along the y axis can be neglected.

(9) $$V_{x}\frac{\partial T}{\partial x}=\chi \left(\frac{\partial^{2}T}{\partial x^{2}}+\frac{\partial^{2}T}{\partial z^{2}}\right)$$

The boundary conditions for this case can be written as:

(10) $${-\lambda \frac{\partial T}{\partial z}|}_{z=0}=q\left(x\right),{T|}_{z\to \infty }\to T_{0}$$

where λ and χ correspond to heat conductivity and thermal diffusivity coefficients, respectively; *q*(*x*) is the distribution of the total energy flow on the melt pool surface; and *T*_0_ is the initial temperature of the substrate.

Since the energy flux density on the pool surface includes the laser radiation intensity *I* and the convective heat flux brought by the heated powder, we can write:

(11) $$q(x)=I(x)\cdot A+j(x)\cdot c\cdot \left(T_{p}(x)-T_{0}\right)$$

To determine *T_p_*, we can use a well-known analytical solution to the problem of temperature distribution in a homogeneous ball of radius *R* with an initial temperature *T*_0_ for the case when a constant heat flow *q_p_* is fed into the ball through its surface [16,17]:

$$T_{i}(r,t)=T_{0}+\frac{q_{p}R}{\lambda }\left(\frac{3\chi t}{R^{2}}-\frac{3R^{2}-5r^{2}}{10R^{2}}\right)-\frac{2q_{p}}{\lambda Rr}\sum_{i=1}^{\infty }\frac{\sin\left(\mu_{i}r\right)}{\mu_{i}^{3}\cos\left(R\mu_{i}\right)}e^{-\chi \mu_{i}^{2}t}$$

where *μ_n_* are the positive roots of the equation tg(Rμ)=Rμ; and *t* is the heating time, which is determined for each particle as the time of flight through the laser radiation zone before it enters the melt pool. Knowing the density of the powder flow in the gas–powder jet, the trajectory of the particles, their size, and the amount that got into the melt pool—for example, from [18]—it is possible to obtain the temperature distribution *T_p_*(*x*) in the gas-–powder jet at the time of meeting with the melt surface.

Furthermore, using the method of solving the convective heat transfer equation described in [6], for the temperature field during surfacing of the i-th layer, we obtain:

(12) $$T\left(x,z\right)=\frac{e^{-\frac{Vx}{2\chi }}}{\lambda }\int_{-\infty }^{\infty }\left(A\cdot I(x^{\prime })+j(x^{\prime })c\left(T_{p}(x^{\prime })-T_{0}\right)\right)e^{-\frac{Vx^{\prime }}{2\chi }}K_{0}\left(\frac{V}{2\chi }\sqrt{z^{2}+{\left(x-x^{\prime }\right)}^{2}}\right)dx^{\prime }+T_{w}$$

where *T_w_* is the residual temperature of the previous layer when applying the bead, determined by the product construction strategy.

## 3. Results and Discussion

The proposed model was tested at the Institute of Laser and Welding Technologies of St. Petersburg State Marine Technical University, for various products and materials. A comparison of the calculation results and experimental data showed that the error does not exceed 20%; this is a good indicator of the performance of a fast, semi-analytical model.

For the calculations, data on physical and optical properties of the works [19,20,21,22,23,24,25,26] were used. The data shown in Table 1 were averaged.

**Figure 2:**
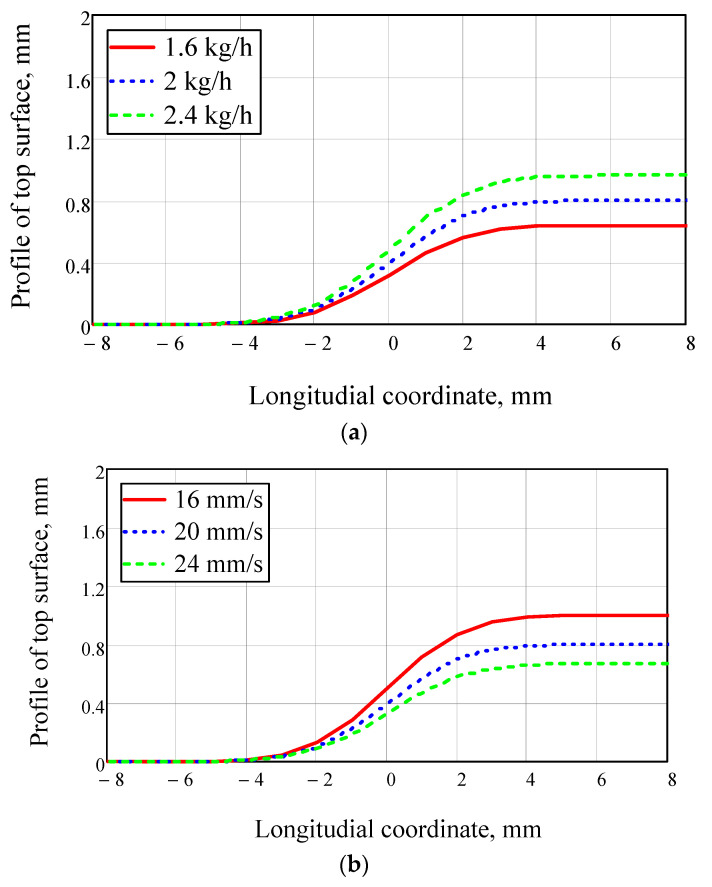
Melt pool top surface profile for 316 L steel for different powder feed rates (**a**) and motion speeds (**b**); laser beam radius on the surface was 2.5 mm, beam power was 2000 W, and powder jet diameter was 3 mm.

Examples of surface profile calculations for different values of motion speed and powder feed rate are shown in Figure 2. It is evident that the surface profile depends on the melt pool length “*L*” and depth “*H*”.

**Figure 3:**
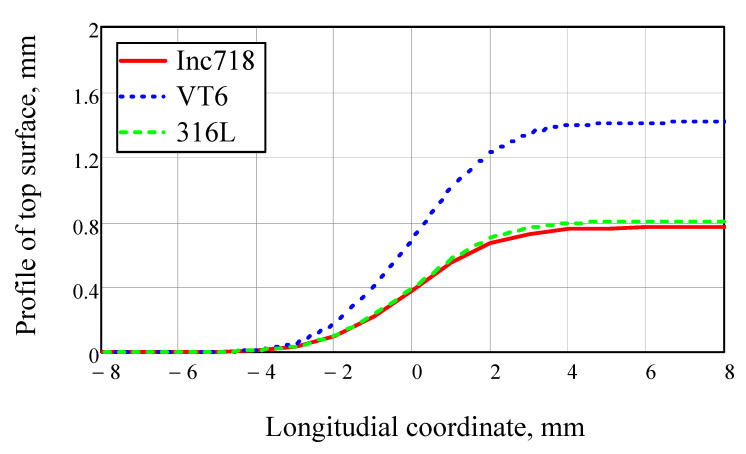
Melt pool top surface profiles for Inconel718, VT6, and 316 L steel in comparison with one another. Motion speed was 20 mm/s, laser beam power was 2000 W, laser beam radius on the surface was 2.5 mm, powder jet diameter was 3 mm, and powder feed rate was 2 kg/h.

A comparison of the melt pool surface profiles for different materials is shown in Figure 3. It can be seen from the figure that with the same mode parameters, the VT6 alloy gives a greater increment in height when the bead is deposited. At the same time, the process efficiency for steel and nickel alloy is approximately the same.

**Figure 4:**
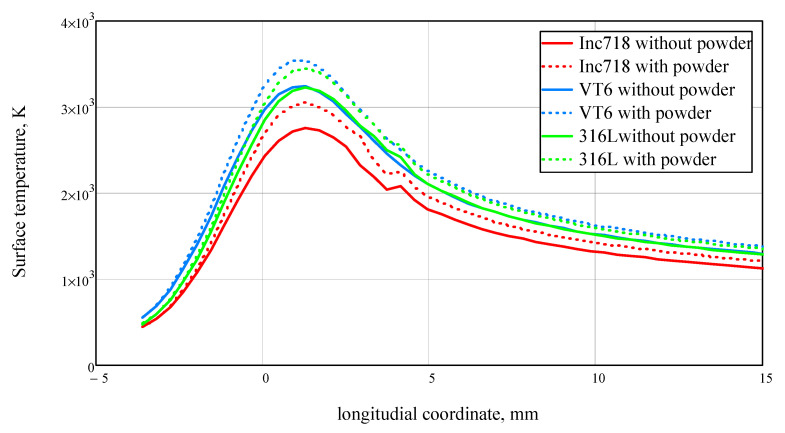
Temperature distribution along the melt pool length. Laser power was 2000 W, motion speed was 20 mm/s, laser beam radius on the surface was 2.5 mm, powder jet diameter was 3 mm, and powder feed rate was 2 kg/h.

The impact of the heated powder jet on the surface temperature distribution along melt pool surface is shown in Figure 4.

**Figure 5:**
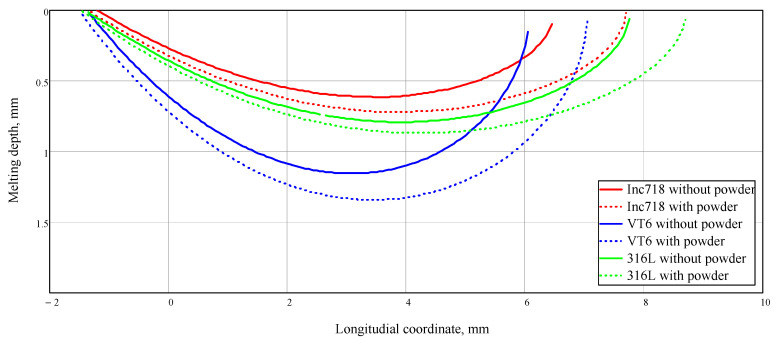
Melt pool shape. Laser power was 2000 W, motion speed was 20 mm/s, laser beam radius on the surface was 2.5 mm, powder jet diameter was 3 mm, and powder feed rate was 2 kg/h.

Calculations show that even a small addition of energy flux with the heated powder leads to an increase in the melt pool’s size (Figure 5).

For all materials, the contribution of heat from the heated powder was significant. In addition, due to the greater absorption capacity of the titanium alloy, not only was the melt pool length increased, but also the depth. For Inconel 718 and 316 L steel, this effect was not so significant. The figure shows that for 316 L steel and Inconel 718 alloy, there is a more elongated, but shallow melt pool, while for titanium alloy, on the other hand, there is a shorter and deeper melt pool. In the presence of a smaller pool, the amount of powder entering the melt will be less, and the efficiency of the process will decrease. It turns out that despite the higher absorption coefficient, having a melt pool with a smaller surface area, the efficiency of the deposition process for titanium alloy may be less than expected. This fact should be taken into account when selecting processing modes.

## 4. Conclusions

Direct laser deposition is a complex physical process. When developing new modes, a number of experiments can be replaced by a physically adequate mathematical modeling. If possible, the mutual influence of different processes on one other should be taken into account. The hydrodynamics of the melt are inextricably connected to the temperature field formed in the substrate under the action of laser radiation. In addition to the radiation itself, the final values of the temperature field in general, and the surface temperature in particular, are significantly affected by the heated powder entering the melt via a gas–powder jet. The effect of additional heat input from the powder on the pool length is noticeable for all of the materials considered—the simulation results clearly demonstrated this. Such an increase can be predicted with high probability for any metal materials. The quantitative contribution of heat from the powder depends on both the thermophysical and optical properties of the material. For some materials, it may be significant to increase not only the length of the melt pool, but also its depth—as, for example, it turned out to be for a titanium alloy. However, it should be borne in mind that reducing the size of the pool at the same mass flow density will lead to a decrease in the efficiency of the deposition process. With a significant variability in the properties of materials, the choice of parameters should mainly be carried out using mathematical modeling, since the experimental selection of modes can be extremely time-consuming.

## Figures and Tables

**Table 1:** Thermophysical and optical properties of the chosen materials.

Properties	Inconel 718	VT6	316 L
Heat capacity, J/(G·K)	0.435	0.546	0.45
Heat conductivity W/(m·K)	8.9	26	30
Density, kg/m^3^	8190	4430	7800
Melting point, K	1600	1920	1710
Reflectivity for 1.06 μm, %	77	61	68

## Data Availability

The data presented in this study are available on request from the corresponding author.

## Nomenclature

*L*	Melt pool length, mm
*H*	Melt pool depth, mm
*b*	Melt pool half-width, mm
*R*	Curvature radius of a bead profile
*V*	Laser beam motion speed, mm/s
*V_m_*(*x*,*y*,*z*)	Liquid metal flow velocity, mm/s
*V_x_*, *V_y_*, *V_z_*	X, Y, and Z components of the liquid metal flow velocity, mm/s
*c*	Heat capacity, J/(kg·K)
*λ*	Heat conductivity, W/(m·K)
*χ*	Thermal diffusivity, m^2^/s
*ρ*	Density, kg/m^3^
*ν*	Kinematic viscosity of the liquid melt, m^2^/s
*η*	Dynamic viscosity of the liquid melt, Pa·s
*α*, *β*, *γ*	Coefficients of the parabolic equation of the melt velocity X component
*σ*, *σ**	Surface tension, N/m
*T*_0_	Initial temperature, environment temperature, K
*T_m_*	Melting point, K
*T_b_*	Boiling point, K
*T_s_*	Maximum surface temperature, K
*T_w_*	Residual temperature of the previous deposited bead, K
*p*	Pressure, Pa
*p_add_*	Additional pressure over change of the longitudinal curvature radius, Pa
*j*(*x*)	Powder mass flow density, kg/(s·m^2^)
*q*(*x*)	Distribution of the total energy flux on the melt pool surface, W/m
*I*(*x*)	Intensity of the laser beam radiation, W/m
*A*	Light energy absorption coefficient
*T_i_*(*r*,*t*)	Temperature of a particle at co-ordinate r and heating time t, K
*T_p_*(*x*)	Temperature distribution in the powder jet along the X axis, K
